# A novel parvovirus (family *Parvoviridae*) in a freshwater fish, zander (*Sander lucioperca*)

**DOI:** 10.1007/s00705-022-05419-5

**Published:** 2022-03-12

**Authors:** Gábor Reuter, Ákos Boros, Róbert Mátics, Eda Altan, Eric Delwart, Péter Pankovics

**Affiliations:** 1 Department of Medical Microbiology and Immunology, Medical School, University of Pécs, Szigeti út 12., 7624 Pécs, Hungary; 2 Hungarian Nature Research Society, Ajka, Hungary; 3 Vitalant Research Institute, San Francisco, CA USA; 4 University of California, San Francisco, CA USA

## Abstract

In this study, a novel parvovirus (zander/M5/2015/HUN, OK236393) was detected in faecal specimens from a fish – zander or pikeperch (*Sander lucioperca*) – and genetically characterized using viral metagenomics and PCR methods. The NS1 and VP1 proteins of zander/M5/2015/HUN share <30% aa sequence identity, respectively, with the corresponding proteins of known members of the family *Parvoviridae.* Out of 62 faecal specimens collected from 13 freshwater fish species, three (4.8%) samples were positive by PCR for the novel parvovirus – all from zander. This is the second parvovirus detected in fish – after the disease-causing tilapia parvovirus of the subfamily *Hamaparvovirinae* – and it potentially represents a novel genus in the subfamily *Parvovirinae*.

**Supplementary Information:**

The online version contains supplementary material available at 10.1007/s00705-022-05419-5.

Parvoviruses are non-enveloped, genetically diverse viruses with a 4- to 6-kb-long single-stranded DNA genome, which generally contains only two major open reading frames (ORFs). The non-structural (NS) region encodes the enzymes used for replication, and the structural (VP) region encodes the capsid protein. Both ends of the genome contain hairpin-like untranslated genome termini [3]. The family *Parvoviridae* contains three subfamilies: the *Densovirinae*, whose members infect invertebrates, and the *Parvovirinae* and *Hamaparvovirinae*, whose members infect vertebrates [3, 10]. The subfamilies *Densovirinae*, *Parvovirinae*, and *Hamaparvovirinae* have 11, 10, and 5 genera, respectively ([10], https://talk.ictvonline.org/taxonomy/).

Parvoviruses are capable of infecting a wide range of animals from insects to mammals. However, the first parvovirus from fish was discovered only in 2019 [6]. Tilapia parvovirus was identified in intestinal samples of tilapia (*Oreochromis niloticus*) and faecal samples of crocodiles fed with tilapia using next-generation sequencing (NGS) in Hainan, China [6]. Subsequently, the same parvovirus was reported in a high-mortality outbreak affecting adult farmed Nile tilapia in Hubei, China [8], and red hybrid tilapia in Thailand [14]. Tilapia parvovirus belongs to the genus *Chaphamaparvovirus,* subfamily *Hamaparvovirinae*. The only other parvovirus that has been identified in fish is syngnathid ichthamaparvovirus 1 (genus *Chaphamaparvovirus,* subfamily *Hamaparvovirinae)*, which was sequenced and identified through NGS assemblies from a tissue homogenate of a gulf pipefish (*Syngnathus scovelli*) [9]. These pioneering studies indicate that fish could be unexplored but important hosts for parvoviruses.

In this study, we report a member of a potentially new genus of parvovirus detected in faecal samples from a freshwater fish – zander or pikeperch (*Sander lucioperca*) – in Hungary.

A total of 62 faecal samples were collected directly from 13 different species of freshwater fish (Table 1) living in natural and artificial open-air fishponds in the vicinity of the town of Szarvas (East Hungary) in 2015 [7]. The fish showed no clinical signs of disease during the sample collection and were released immediately after sampling.

**Table 1:** PCR detection of zander parvovirus in faecal specimens and distribution in different freshwater fish species

Fish species	No. of samples tested	No. of samples positive (%)
Freshwater bream (*Abramis brama*)	13	0
Blue bream (*Ballerus ballerus*)	10	0
European perch (*Perca fluviatilis*)	8	0
Zander (*Sander lucioperca*)	7	3 (42.8%)
White bream (*Blicca bjoerkna*)	5	0
Roach (*Rutilus rutilus*)	5	0
Black bullhead (*Ameiurus melas*)	4	0
Volga pikeperch (*Sander volgensis*)	3	0
Prussian carp (*Carassius auratus gibelio*)	3	0
White-eyed bream (*Abramis sapa*)	1	0
European carp (*Cyprinus carpio*)	1	0
Sabre carp (*Pelecus cultratus*)	1	0
Silver carp (*Hypophthalmichthys molitrix*)	1	0

A specimen pool containing faecal samples from three zander (*Sander lucioperca*) (M5, M6, and M8) were selected for viral metagenomics analysis. Briefly, 200 μl of PBS-diluted specimen was passed through a 0.45-μm sterile filter (Millipore) and centrifuged at 6,000 × *g* for 5 min. Then, the filtrate was treated with a mixture of DNases and RNases (Turbo DNase, Invitrogen; Baseline Zero DNase, Epicentre Biotechnologies; Benzonase Nuclease, Novagen; RNase A, Fermentas) at 37 °C for 2 hours to digest unprotected nucleic acids [11]. Virus-particle-protected nucleic acids were extracted using the QIAamp spin-column technique (QIAamp Viral RNA Mini Kit, QIAGEN) using an RNase inhibitor (RiboLock RNase Inhibitor, Fermentas) at the elution step. Sequence-independent random RT-PCR amplification [15] with 20 PCR cycles was used, and a 250-bp paired-end viral cDNA library was constructed using a Nextera XT DNA Library Preparation Kit (Illumina). The library was sequenced on a MiSeq Illumina platform according to the manufacturer’s instructions. The resulting metagenomic reads were trimmed, assembled *de novo* [5], and analyzed using an *in-house *pipeline [11]. Briefly, singlets and the assembled contigs greater than 250 bp in length were compared to the GenBank [2] protein database using BLASTx (version 2.2.7) [1] using an E-value cutoff of 0.01. Candidate viral hits were then compared to a non-virus non-redundant protein database to remove false-positive viral hits. Virus-family-level categorization of all viral metagenomic sequences was based on the best BLASTx scores (E-value ≤ 10^-10^). All of the genomic data have been deposited in NCBI BioProject PRJNA784176. Raw reads are available in the Sequence Read Archive (SRA) database under accession number SAMN23475123.

A sequence-specific screening primer pair (ZanderParvo-screen-F, GGC TAA TCA TCA AAC AGG AAA GA; ZanderParvo-screen-R, AGC TCC CAC CAC TTA ATA TCT T) was designed to identify a 492-nucleotide-long portion of the NS1 region of the viral genome of the study strain from the specimen pool. The PCR thermocycler program consisted of 1 min at 95ºC, 40 cycles for 30 s at 95ºC, 10 s at 48ºC, and 30 s at 72ºC, and a final 10-min extension at 72ºC, using a C1000 Touch Thermal Cycler (Bio-Rad). In addition, different sets of specific primers were designed based on the sequences of the metagenomics reads/contigs and the amplified PCR products for verification of the metagenomics contig by Sanger sequencing (using a BigDye Terminator v1.1 Cycle Sequencing Kit [Thermo Fisher] on an ABI3500 Genetic Analyzer [Applied Biosystems, Hitachi, Tokyo, Japan]) and to obtain the nearly complete viral genome of the study strain. Faecal samples from each fish were tested individually by the PCR method, using the ZanderParvo-screen-F/R screening primer-pair.

ClustalX (version 2.1) and GeneDoc (version 2.7) were used to align the corresponding amino acid sequences of the helicase domain of NS1 from this study and those of representative prototype parvoviruses of the subfamily *Parvovirinae* [10]. For the construction of an NS1-helicase phylogenetic tree, the BEAST v. 1.10.4 software package was used with a setup similar to that described by Pénzes et al. [10]. Briefly, the substitution model LG+I+G+F with a lognormal relaxed clock and Youle process was used throughout 10 million generations.

Nucleotide (nt) and amino acid (aa) sequences of zander parvoviruses (zander/M5/2015/HUN [near-complete genome], zander/M3/2015/HUN [partial, NS1/VP1 joining region], and zander/M7/2015/HUN (partial, NS1/VP1 joining region]) have been deposited in the GenBank database under accession numbers OK236393-OK236395.

A specimen pool containing three faecal samples from zander was subjected to viral metagenomics analysis. After *de novo* assembly of the 33,797,786 sequence reads, 161,647 reads showed similarity (BLASTx cutoff E-value ≤ 10^-10^) to viral sequences. The detected sequences containing more than 100 reads were from viruses of the families *Picornaviridae* (n = 87,506, see reference [7]), unclassified viruses (n = 31,825), *Parvoviridae* (n = 19,998), *Circoviridae* (n = 6,346), *Tombusviridae* (5,535), *Microviridae* (n = 3,532), *Dicistroviridae* (n = 1,388), *Virgaviridae* (n = 1,090), *Phycodnaviridae* (n = 833), *Geminiviridae* (n = 714), *Nanoviridae* (n = 585), *Siphoviridae* (n = 360), *Podoviridae* (n = 358), *Myoviridae* (n = 270), *Alphaflexiviridae* (n = 199), and *Herpesviridae* (n = 159).

Sequence reads/contigs corresponding to members of the family *Parvoviridae* were selected for further analysis. The parvovirus sequences matched best to members of an unassigned parvovirus genus (n = 14,994 reads) and nine parvovirus genera (*Protoparvovirus* [n = 1,103], the former genus *Ambidensovirus* [n = 1100], *Bocaparvovirus* [n = 925], *Dependoparvovirus* [n = 658], *Tetraparvovirus* [n = 604], *Iteradensovirus* [n = 453], *Aveparvovirus* [n = 104], *Erythroparvovirus* [n = 43], and *Amdoparvovirus* [n = 14]). A sequence-specific screening primer-pair was designed based on the NS1 region of the longest parvovirus sequence contig to identify the parvovirus strain from the specimen pool. One of the three specimens from zander was PCR-positive, and this sample (zander/M5/2015/HUN) was selected for further study.

The continuous parvoviral genome sequence was determined and verified using different sets of specific primers designed based on the sequences of the metagenomics reads/contigs and the amplified PCR products by Sanger sequencing. The nearly complete genome sequence – including the complete coding regions – of the zander parvovirus (zander/M5/2015/HUN) is 4,322 nucleotides long (Fig. 1) and contains two well-known parvovirus ORFs (NS1 and VP1). The NS1 (replicase) region is 1,956 nucleotides (651 aa) in length. It encodes the helicase, including the conserved ATP- or GTP-binding Walker A loop aa motif (GxxxxGKT/S; _355_**G**PPST**GKT**_362_), and the Walker B (xxxxEE; _395_LIWM**EE**_400_), Walker B’ (KxxxxGxxxxxxxK; _412_**K**GVTG**G**TKIRVDK**K**_425_), and Walker C (PIxIXXN; _437_**P**LVWTT**N**_443_) aa motifs [17]. In addition, the NS1 protein contains two conserved replication initiator (endonuclease) motifs, xxHuHxxxx (DH_109_**H**M**H**_111_VIIP) and YxxxK (_183_**Y**FSK**K**_187_) (conserved aa are indicated in bold letters, and “u” as a hydrophobic residue) [13, https://talk.ictvonline.org/ictv-reports/ictv_online_report/ssdna-viruses/w/parvoviridae]). The NS1 protein shares 28.4% and 27.6% aa sequence identity (query coverage: 58% and 58%, respectively) with the corresponding NS1 proteins of murine adeno-associated virus 1 (NC_055485) from mice [18] and a dependovirus (MT138242) found in anal swabs of birds [Xiao et al., unpublished], both belonging to the genus *Dependoparvovirus,* subfamily *Parvovirinae*, as the closest matches by BLASTp. The VP1 was predicted to be 582 aa (1,749 nt) in length, which is similar to those of other members of the subfamily *Parvovirinae* (537-781 aa). The N-terminal glycine-rich region is present (34 glycine residues – 28.5% - in the first 119 aa of VP1) as is the GPGN calcium-binding loop (_35_**GPGN**_38_) [16], but the phospholipase A_2_ (PLA_2_) catalytic residues (DxxAxxHDxxY + D), which are widely present in the VP1 unique part (VP1up) of many parvoviruses [16] were not identifiable in the VP1 proteins of zander/M5/2015/HUN. Interestingly, no similar VP1 protein sequence was found in GenBank using BLASTp. NS1 overlaps by 361 nt with the VP1 region, and this was confirmed by PCR and sequencing of three different strains (M3, M5, and M7). The left and right terminal sequences of the genome were partially determined. The 3’ genome end is more than 475 nt long in zander/M5/2015/HUN. Similar nucleotide sequences were not found in the GenBank database. The 5’ genome end could be more than 503 nt long; however, this region potentially encodes a 125-aa-long protein (Fig. 1). Similar nucleotide and protein sequences were not found in GenBank.

**Fig. 1:**
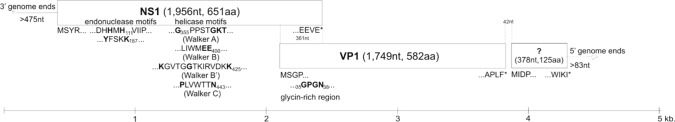
Genome organization of the parvovirus zander/M5/2015/HUN (OK236393). The 4-aa-long N-and C-terminal protein ends and the conserved aa motifs of the coding regions (NS1 and VP1 and a potential unknown protein at the 5’ genome end) are indicated. Bold capital letters indicate conserved amino acids in the NS1 endonuclease and helicase motifs. A star (*) indicates a stop codon. The nt and aa length of each coding region is shown.

Comparing the 4,322-nt-long study sequence to the metagenomic reads/contigs corresponding to members of the family *Parvoviridae* (N = 19,998) in the specimen pool, 95.38% of these metagenomic sequences could be aligned to the nt sequence of zander/M5/2015/HUN, indicating that the vast majority of these parvovirus sequences represent one parvovirus strain.

Phylogenetic analysis based on aa sequences of the tripartite helicase domain of NS1 showed that zander/M5/2015/HUN formed a distinct lineage – potentially representing a new genus – in the subfamily *Parvovirinae* (Fig. 2).

**Fig. 2:**
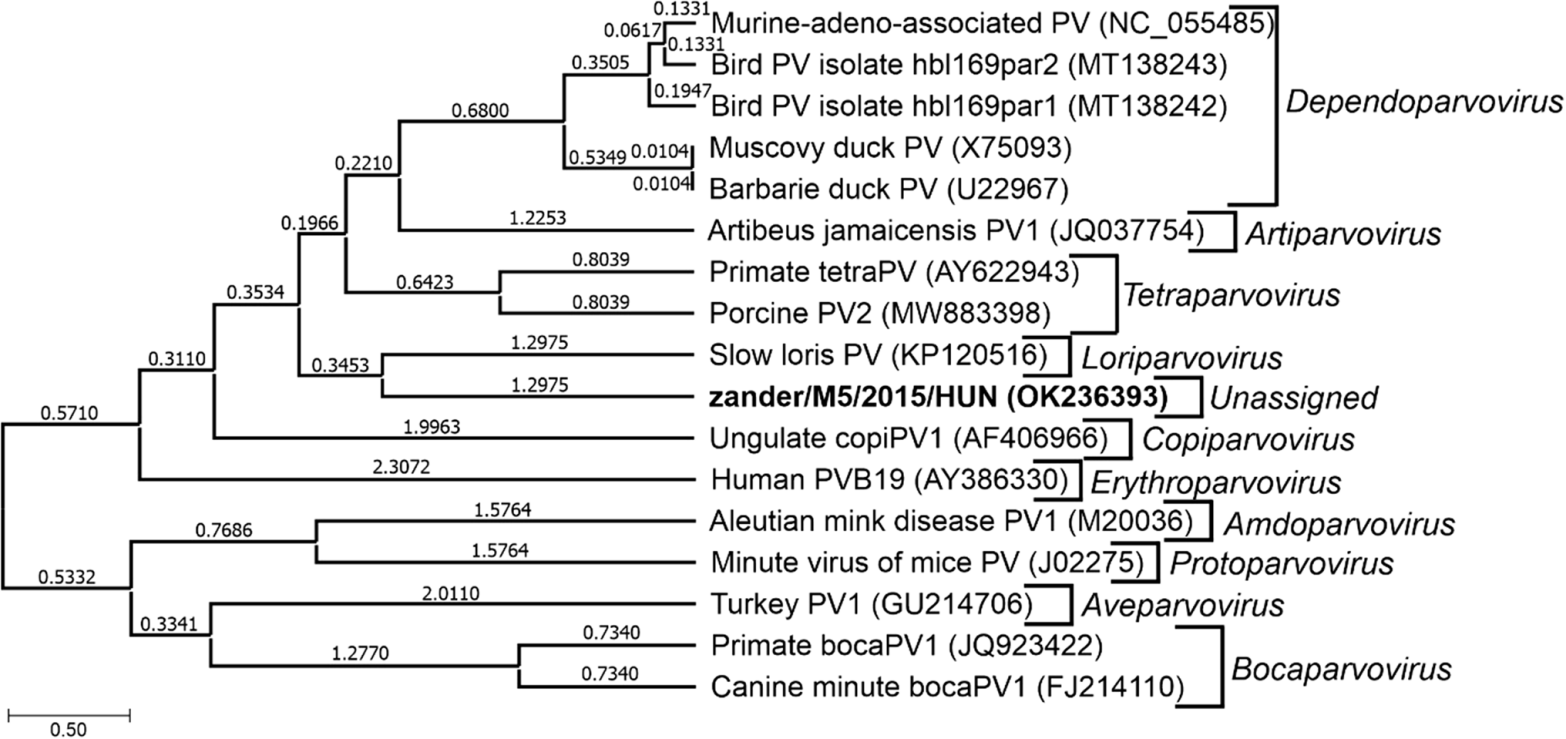
Phylogenetic analysis of zander/M5/2015/HUN (OK236393, bold letters) and representatives of 10 genera in the subfamily *Parvovirinae* based on the ~460-aa-long tripartite helicase domain of NS1. The dendrogram was constructed based on an amino acid sequence alignment of tripartite helicase domains by Bayesian Evolutionary Analysis Utility version v1.10.4 (BEAST) [12] using the LG+I+G+F substitution model, a lognormal relaxed clock, and Youle process, throughout 10,000,000 generations. The tree is drawn to scale with branch lengths measured in the average number of substitutions per time unit. PV = parvovirus

Applying the screening primer pairs, three (4.8%) of the 62 specimens (M3, M5, and M7) were PCR-positive for the study strain, all three of which were from zander (*Sander lucioperca*) (3 out of 7 specimens, 42.8%) (Table 1). The nucleotide sequences of the 995-nt-long NS1/VP1 joining region were 100% and 99.6% identical (2 synonymous nt mutations) between zander/M5/2015/HUN (OK236393) and zander/M7/2015/HUN (OK236395) and between zander/M5/2015/HUN and zander/M3/2015/HUN (OK236394), respectively.

The number of known members of the family *Parvoviridae* is rapidly expanding [10]. At present, there are 26 parvovirus genera, and their members have been discovered in a wide range of animal host species from insects to humans. In spite of this, the first fish-origin parvovirus (a disease-causing tilapia parvovirus from an intensively aquafarmed tilapia fish [6] for human consumption) was not reported until 2019.

This study represents the second detection and characterization of a novel parvovirus from faecal specimens of a freshwater fish, zander. According to the species demarcation criteria of the International Committee on Taxonomy of Viruses (ICTV) *Parvoviridae* Study Group, two parvoviruses have to share >85% aa sequence identity in the NS1 protein to belong to the same species [4]. In addition, all parvoviruses in a genus should be monophyletic and encode NS1 proteins that are >30% identical to each other at the amino acid sequence level [4]. Following these rules, since the parvovirus from zander described here has less than 30% aa identity in the NS1 protein to any presently known parvovirus NS1 proteins, it potentially represents a new genus and a new species in the family *Parvoviridae*. While the tilapia parvovirus is a member of the subfamily *Hamaparvovirinae*, the novel parvovirus from zander belongs to another parvovirus subfamily, *Parvovirinae*.

Although this parvovirus was identified in faecal samples from zander, the host from which this novel virus originated remains unknown. While we could detect it in more than one faecal specimen from zander, we cannot exclude the possibility of a dietary origin of the virus. The pathogenicity and impact of this novel virus on aquafarming should be investigated in further studies, especially in the light of the fact that tilapia parvovirus causes disease in fish [6, 8]. It should also be noted, that one of the parvovirus-positive faecal specimen (M7) from zander also contained a potentially novel fish-origin picornavirus (family *Picornaviridae*) [7] as a co-infection.

Systematic investigation of samples collected from aquatic animals is necessary to explore the genetic diversity of fish-origin parvoviruses and to identify potential disease-causing pathogens.

## Supplementary Information

Below is the link to the electronic supplementary material.

Supplementary file1 (TXT 9 kb)

## Declarations

### Web pages

Virus Taxonomy: 2020 Release, International Committee on Taxonomy of Viruses (ICTV) March 2021, MSL ♯36, https://talk.ictvonline.org/taxonomy/; International Committee on Taxonomy of Viruses (ICTV) *Parvoviridae*: https://talk.ictvonline.org/ictv-reports/ictv_online_report/ssdna-viruses/w/parvoviridae.
